# Identification and Monitoring of Host Cell Proteins by Mass Spectrometry Combined with High Performance Immunochemistry Testing

**DOI:** 10.1371/journal.pone.0081639

**Published:** 2013-11-27

**Authors:** Katrin Bomans, Antje Lang, Veronika Roedl, Lisa Adolf, Kyrillos Kyriosoglou, Katharina Diepold, Gabriele Eberl, Michael Mølhøj, Ulrike Strauss, Christian Schmalz, Rudolf Vogel, Dietmar Reusch, Harald Wegele, Michael Wiedmann, Patrick Bulau

**Affiliations:** 1 Pharma Development, Roche Diagnostics GmbH, Penzberg, Germany; 2 Pharma Biotech, Roche Diagnostics GmbH, Penzberg, Germany; 3 Professional Diagnostics, Roche Diagnostics GmbH, Penzberg, Germany; New York University, United States of America

## Abstract

Biotherapeutics are often produced in non-human host cells like Escherichia coli, yeast, and various mammalian cell lines. A major focus of any therapeutic protein purification process is to reduce host cell proteins to an acceptable low level. In this study, various *E. coli* host cell proteins were identified at different purifications steps by HPLC fractionation, SDS-PAGE analysis, and tryptic peptide mapping combined with online liquid chromatography mass spectrometry (LC-MS). However, no host cell proteins could be verified by direct LC-MS analysis of final drug substance material. In contrast, the application of affinity enrichment chromatography prior to comprehensive LC-MS was adequate to identify several low abundant host cell proteins at the final drug substance level. Bacterial alkaline phosphatase (BAP) was identified as being the most abundant host cell protein at several purification steps. Thus, we firstly established two different assays for enzymatic and immunological BAP monitoring using the cobas® technology. By using this strategy we were able to demonstrate an almost complete removal of BAP enzymatic activity by the established therapeutic protein purification process. In summary, the impact of fermentation, purification, and formulation conditions on host cell protein removal and biological activity can be conducted by monitoring process-specific host cell proteins in a GMP-compatible and high-throughput (> 1000 samples/day) manner.

## Introduction

Host cell proteins (HCPs) carry potential clinical safety risks for patients treated with biologics. On the one hand, HCP might cause an immune response (due to their “non-self” nature), adjuvant activity, and theoretically also function in the human body [1–3]. Furthermore, HCPs with protease activity have the potential to impact product stability [4]. Consequently, regulatory guidelines mandate the setting of HCP specifications [5].

Thus, one key aspect of any biologics manufacturing is to reduce HCP to levels considered acceptable in the final drug [6]. The HCP composition is impacted by the proteome complexity of the utilized host expression system [7–9], the manner in which the therapeutic protein is expressed [10–13], and the purification process itself [10,12]. Moreover, all methods for analytical HCP characterization face challenges due to the dynamic range of HCP abundance at proteome and final drug level.

Several analytical techniques have been used for the detection, identification, and quantification of HCPs [1,3,14,15]. To perform bio-process and release analytics, immunoassays like protein gel blots and multicomponent generic or process-specific enzyme-linked immunosorbent assays (ELISA) are most commonly used to detect and monitor HCPs [16–18]. The ELISA technique is typically applied for HCP analysis, mostly due to the good precision of the method and also that it provides quantitative results for setting control limits and specifications. However, generic ELISAs do not offer complete coverage for all process-specific HCPs and process-specific ELISAs might be not qualified to evaluate the HCP content after process changes [3,16–18]. Two-dimensional gel electrophoresis combined with fluorescent staining is also applied for the detection and quantification of HCPs [19,20]. The technique is semi-quantitative, has a limited dynamic range, and needs mass spectrometry for HCP identification. 

Approaches involving liquid chromatography coupled to mass spectrometry (LC-MS) provide alternative solutions for product characterization within the biopharmaceutical industry [21–25].. Advances in two dimensional LC-MS (2D–LC-MS) have enabled the analysis of low-abundance analytes in complex protein mixtures [26–28]. Recently, the identification and quantification of HCPs in biotherapeutics by 2D–LC-MS was demonstrated [29,30]. 

In the present study, an approach employing affinity chromatography to capture HCPs, highly sensitive LC-MSMS, and high throughput immunoassay testing for the enrichment, identification and quantification of HCPs in biotherapeutics was developed. This test system allowed us to identify and monitor Bacterial Alkaline Phosphatase in a biopharmaceutical purification process.

## Results

**Figure 1:**
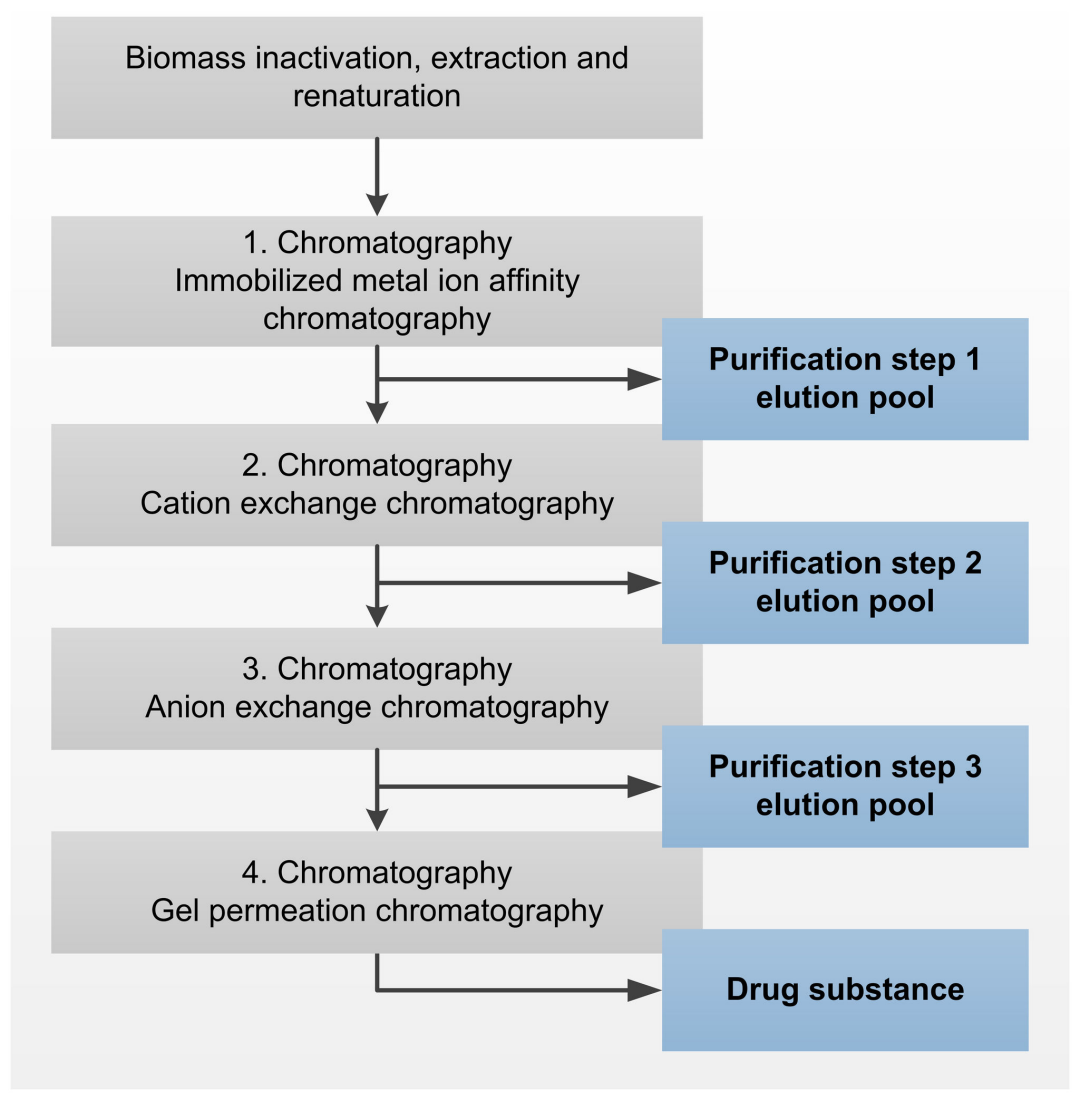
Scheme of the investigated protein purification processes.

**Figure 2:**
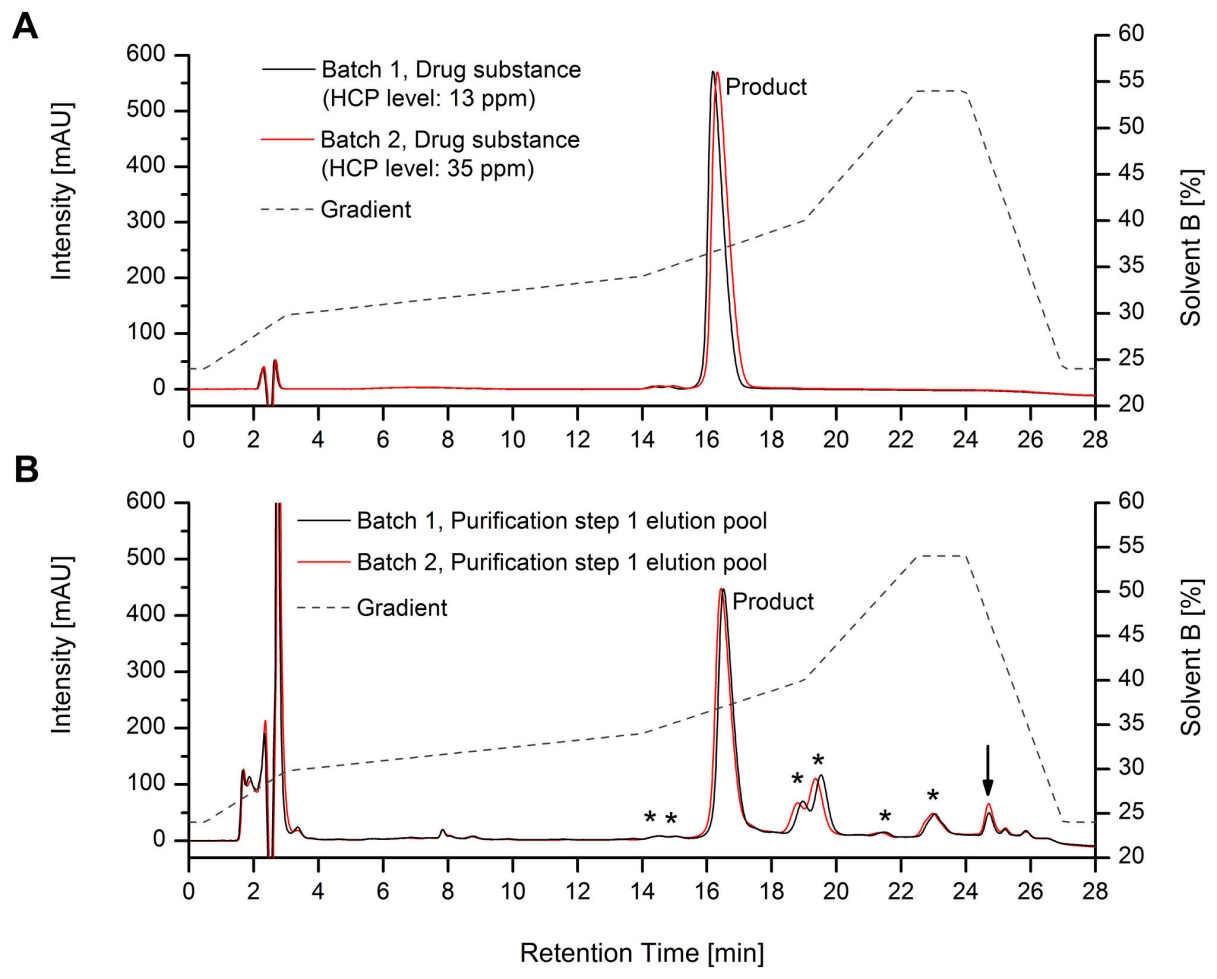
Monitoring of product variants (*) by RP-HPLC. Batches with different HCP content at drug substance level (A) and the product elution pool after metal affinity purification step 1 (B). Batch differences are marked by an arrow.

Increased levels of HCPs were detected in the manufacturing process for a recombinant protein derived from *E. coli* by ELISA and RP-HPLC analysis. At final drug substance level, several batches with HCP levels minimal greater than the release specification of 30 ppm were observed by a process-specific ELISA system. RP-HPLC analysis with UV detection is routinely applied to monitor product variants at the final drug substance level and at various purifications steps (Figure 1). At the final drug substance level no significant differences in product purity where observed for batches with elevated HCP levels (Figure 2A). The first chromatographic purification step of the recombinant protein is accomplished by metal chelate chromatography (purification step 1). At this stage several product variants (marked by asterisks) can be observed in the obtained elution pool (Figure 2B). 

**Figure 3:**
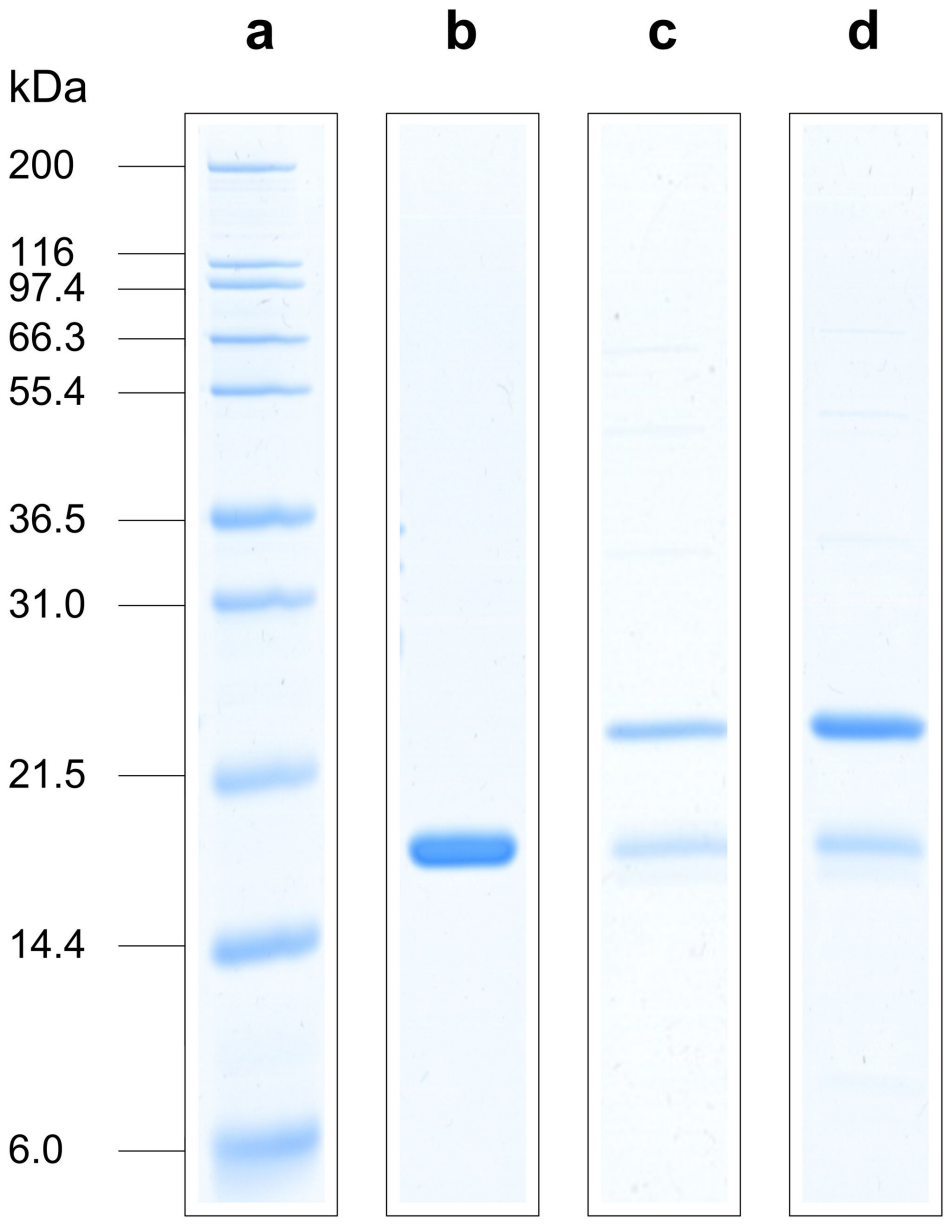
SDS-PAGE analysis of RP-HPLC fractions. (a) Mark12™ Standard; (b) Reference material, drug substance level; (c) RP-HPLC fraction (24-26 min, Figure 2B) of purification step 1 elution pool, HCP content at drug substance level: 13 ppm; (d) RP‑HPLC fraction (24-26 min, Figure 2B) of purification step 1 elution pool, HCP content at drug substance level: 35 ppm.

**Figure 4:**
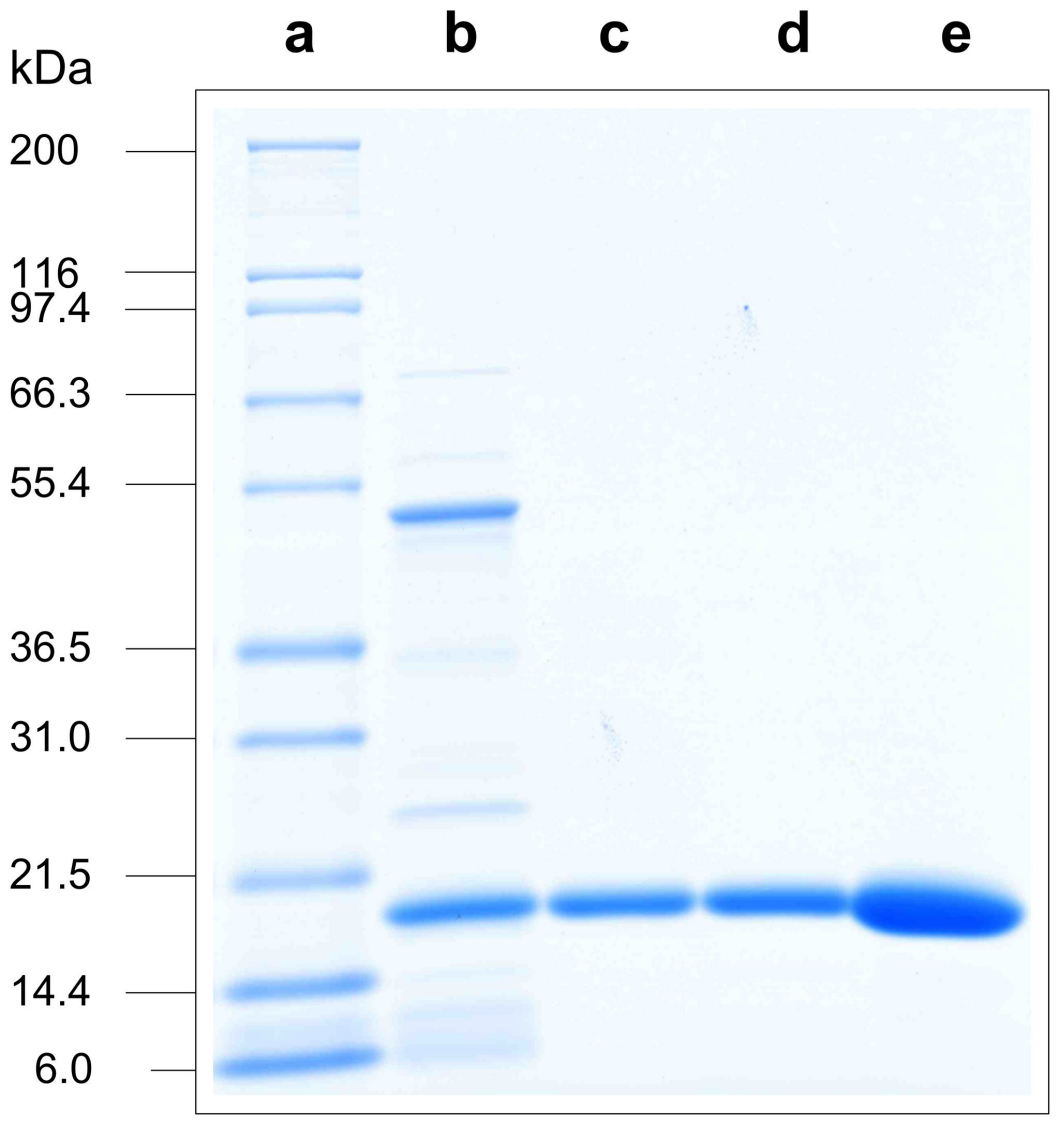
SDS-PAGE analysis of in-process controls. (a) Mark12™ Standard; (b) Purification step 1 elution pool; (c) Purification step 2 elution pool; (d) Purification step 3 elution pool; (e) Drug substance, HCP content: 30 ppm.

In general, no significant differences in product variants where observed for batches with elevated HCP levels at purification step 1 level. However, a slight but distinct increase in peak intensity of the product variant with a retention time of 25 min could be observed for batches with elevated HCP levels (Figure 2B; marked by an arrow). The existence of elevated product variants or HCP levels was further suggested by SDS-PAGE analysis of the respective HPLC fractions (retention time window: 24-26 min), in which an increased content of a protein species with a molecular weight of around 23 kDa was observed for batches with elevated HCP levels (Figure 3). The unknown protein was identified as *E. coli* Alkyl hydroperoxide reductase subunit C (see Table 1) by nano-ESI-MS peptide mapping after in tryptic-gel digestion combined with database searching (mass spectrometric data not shown). Additionally, various potential host cell proteins, not detected by RP-HPLC analysis, were visualized by SDS-PAGE analysis of total purification step 1 elution pool (Figure 4). The dominant HCP with a molecular weight of around 50 kDa was identified as Bacterial Alkaline Phosphatase (BAP; see Table 1) by the procedure as described above (data not shown) and located in the injection peak of the RP-HPLC chromatogram by HPLC fractionation (retention time window: 2-4 min, Figure 2B). The SDS-PAGE analysis of purification step 1-3 elution pools suggest a complete HCP removal by the applied purification process (Figure 4). Nevertheless, tryptic peptide mapping combined with comprehensive online liquid chromatography mass spectrometry (LC-MS/MS) was utilized to identify low abundant HCPs of the different elution pools. Protein identification by database searching was successful to identify various bacterial proteins present in the elution pools of the purifications steps 1 and 2. The identified HCPs are summarized in Table 1. Although the analysis was performed with a highly sensitive LTQ Orbitrap Velos electrospray mass spectrometer, only one bacterial protein (BAP) was verified in the elution pool of purification step 3 and no HCPs were detected at the final drug substance level (Table 1). Since the deployed process-specific ELISA system does demonstrate low abundant HCP levels at final bulk stage (Figure 2), we introduced HCP enrichment by affinity chromatography using the ELISAs polyclonal antibodies in our sample preparation protocol to detect bacterial proteins of low abundance (see details in materials and methods). In order to judge if the HCP enrichment step does significantly enrich or deplete individual HCPs, purification step 1 elution pool was again analyzed with and without applying the affinity enrichment procedure. The identified proteins (identification score ≥ 20; peptides with an FDR <1%) are listed in Table 2 (Repeatability results are summarized in Table S1). All abundant bacterial proteins of the purification step 1 elution pool were also detected after applying the HCP enrichment step. Although the identification scores of some bacterial proteins do suggest alterations of the relative protein abundances only minor effects on protein sequence coverage were observed. In addition, an increasing number of identified HCPs was demonstrated as a consequence of the affinity enrichment procedure. Thus, from a qualitative point of view, the results achieved do not suggest significant alterations of the HCP profile related to the affinity enrichment step. 

**Table 1:** Overview of identified HCPs at different purification (P.) steps.

			P. step 1		P. step 2		P. step 3		Drug substance	
**ACC**	**Identified *E. coli* protein**	MW [kDa]	SC	COV [%]	SC	COV [%]	SC	COV [%]	SC	COV [%]
P00634	Alkaline phosphatase	49.4	**458**	92	**210**	88	**36**	27	*n.d.*	*n.d.*
P0A6Y8	Chaperone protein dnaK	69.1	**250**	80	**151**	61	*n.d.*	*n.d.*	*n.d.*	*n.d.*
P23843	Periplasmic oligopeptide-binding protein	60.9	**214**	78	**107**	68	*n.d.*	*n.d.*	*n.d.*	*n.d.*
P0A6P1	Elongation factor Ts	30.4	**163**	76	**87**	60	*n.d.*	*n.d.*	*n.d.*	*n.d.*
P0A6P9	Enolase	45.6	**116**	69	**70**	50	*n.d.*	*n.d.*	*n.d.*	*n.d.*
P0A877	Tryptophan synthase alpha chain	28.7	**103**	87	**42**	51	*n.d.*	*n.d.*	*n.d.*	*n.d.*
P0AE08	Alkyl hydroperoxide reductase subunit C	20.8	**97**	84	**74**	84	*n.d.*	*n.d.*	*n.d.*	*n.d.*
P0AFM2	Glycine betaine-binding periplasmic protein	36.0	**92**	79	**<20**	20	*n.d.*	*n.d.*	*n.d.*	*n.d.*
P0ABK5	Cysteine synthase A	34.5	**84**	76	**22**	29	*n.d.*	*n.d.*	*n.d.*	*n.d.*
P77395	Uncharacterized protein ybbN	31.8	**68**	58	**52**	62	*n.d.*	*n.d.*	*n.d.*	*n.d.*
	**Total number of identified HCPs (score >20)**		**76**		**16**		**1**		**0**	

Database query was conducted by analyzing LC-MS/MS CID spectra using Proteome Discoverer V1.3 and a false discovery rate FDR < 1%. The Top 10 HCPs were sorted according to the score value at purification step 1 level. ACC, accession number (http://www.uniprot.org/); SC, score; MW, theoretical molecular weight; COV, Sequence coverage; n.d., not detected.

**Table 2:** Comparison of identified HCPs with and without HCP affinity enrichment.

			**Direct analysis**		**After HCP enrichment**	
**ACC**	**Identified *E. coli* protein**	MW [kDa]	SC	COV [%]	SC	COV [%]
P00634	Alkaline phosphatase	49.4	**458**	92	**251**	83
P0A6Y8	Chaperone protein dnaK	69.1	**250**	80	**278**	74
P23843	Periplasmic oligopeptide-binding protein	60.9	**214**	78	**155**	64
P0A6P1	Elongation factor Ts	30.4	**163**	76	**152**	67
P0A6P9	Enolase	45.6	**116**	69	**105**	63
P0A877	Tryptophan synthase alpha chain	28.7	**103**	87	**115**	76
P0AE08	Alkyl hydroperoxide reductase subunit C	20.7	**97**	84	**71**	84
P0AFM2	Glycine betaine-binding periplasmic protein	36.0	**92**	79	**53**	71
P0ABK5	Cysteine synthase A	34.5	**84**	76	**75**	64
P77395	Uncharacterized protein ybbN	31.8	**68**	58	**73**	72
P0A6P7	Probable GTP-binding protein engB	23.5	**65**	68	**78**	77
P0A799	Phosphoglycerate kinase	41.1	**64**	45	**90**	63
P39180	Antigen 43	106.8	**62**	21	**39**	16
P0A862	Thiol peroxidase	17.8	**60**	76	**53**	76
P69913	Carbon storage regulator	6.9	**56**	33	**<20**	33
	**Total number of identified HCPs (score >20)**		**76**		**84**	

Database query was conducted by analyzing LC-MS/MS CID spectra using Proteome Discoverer V1.3 and a false discovery rate FDR < 1% of purification step 1 elution pool samples. The Top 15 HCPs were sorted according to the score value of the direct analysis at purification step 1 level. ACC, accession number (http://www.uniprot.org/); SC, score; MW, theoretical molecular weight; COV, Sequence coverage.

Next, we employed the described approach to identify HCPs at the final drug substance level. A total of 12 bacterial proteins (with an identification score ≥ 20), not detected without affinity enrichment, were identified at final bulk level (Table 3). Eight proteins were already verified at purification step 1 elution pool level (including BAP and Alkyl hydroperoxide reductase subunit C). However, the Protein tolB, DNA protection during starvation protein, 2-amino-4-hydroxy-6-hydroxymethyldihydropteridine pyrophosphokinase, and N-acetylmuramoyl-L-alanine amidase AmiD were only detected at final drug substance level suggesting a relative enrichment of the protein through the purification process.

**Table 3:** Identified HCPs at drug substance level after HCP affinity enrichment.

**ACC**	**Identified *E. coli* protein**	MW [kDa]	SC	COV [%]
P23843	Periplasmic oligopeptide-binding protein	60.9	**93**	60
P0A6Y8	Chaperone protein dnaK	69.1	**77**	40
P23847	Periplasmic dipeptide transport protein	60.3	**77**	46
P00634	Alkaline phosphatase	49.4	**48**	43
P0A855	Protein tolB	45.9	**42**	37
P0ABT2	DNA protection during starvation protein	18.7	**38**	60
P0A6F5	60 kDa chaperonin	57.3	**32**	22
P0AE08	Alkyl hydroperoxide reductase subunit C	20.7	**26**	47
P26281	2-amino-4-hydroxy-6-hydroxymethyldihydropteridine pyrophosphokinase	18.1	**26**	58
P69441	Adenylate kinase	23.6	**22**	36
P75820	N-acetylmuramoyl-L-alanine amidase AmiD	31.1	**21**	30
P0A910	Outer membrane protein A	37.2	**20**	26
	**Total number of identified HCPs (score >20)**		**12**	

Database query was conducted by analyzing LC-MS/MS CID spectra using Proteome Discoverer V1.3 and a false discovery rate FDR < 1%. ACC, accession number (http://www.uniprot.org/); SC, score; MW, theoretical molecular weight; COV, Sequence coverage.

In summary, the application of affinity chromatography combined with comprehensive LC-MS was adequate to identify low abundant HCPs at the final drug substance level. 

Since BAP was identified as being the most abundant HCP at purification step 1 elution pool level and is still traceable at final drug substance level we developed two different assays for enzymatic and immunological BAP monitoring on a cobas INTEGRA® 400 plus and a cobas e 411 system, respectively^1^. The method validation results for both cobas^®^ systems demonstrated acceptable analytical performance and are in accordance with the manufacturer’s accuracy and imprecision criteria (Table 4). The cobas® analysis of all total purification step elution pools and the final drug substance material does demonstrate an almost complete BAP removal by the applied purification process (Table 5). At final drug substance level the detected immunological BAP activity of 0.1 U/mg was close to the quantification limit (0.12 U/mL) and the observed enzymatic BAP activity was below the detection limit of 0.2 mU/mL (see Table 4). As initially described, several batches with increased HCP levels were observed at final drug substance level by a process-specific ELISA system. To verify if BAP does contribute to the elevated total HCP levels the enzymatic and immunological BAP activity of various batches was analyzed at purification step 3 elution pool level. The results are summarized in Table 6 and demonstrate that no causal relationship between elevated total HCP levels and enzymatic and immunological BAP activity is verifiable.

**Table 4:** Analytical performance of the BAP ECLIA and BAP enzymatic activity assay.

Instrument	**cobas e** 411	**cobas INTEGRA® 400 plus**
Assay	BAP ECLIA	BAP Enzymatic activity
Detection Limit [mU/mL]	40	0.2
Quantitation Limit [mU/mL]	120	1.0
Range [mU/mL]	120 - 15 000	1.0 - 1200
Accuracy: Recovery of spike [%]	90 - 118	72 - 105
Precision: Repeatability RSD [%]	< 10	1 - 12
Performance	100 samples/120 min	100 samples/35 min
Dilution of samples	Done by system	Done by system

BAP, Bacterial Alkaline Phosphatase; ECLIA, Electrochemiluminescence Immunoassay.

**Table 5:** Immunological and enzymatic BAP activities at different process levels.

	Protein	HCP ELISA	BAP ECLIA	BAP Enzymatic activity
	*[mg/mL]*	*[ng/mg]*	*[U/mg]*	*[mU/mg]*
Purification Step 1	1.44	n.a.	8390	970
Purification Step 2	0.28	n.a.	38	0.7
Purification Step 3	6.44	264	9.0	0.4
Drug substance	1.74	16	0.1	<DL

BAP, Bacterial Alkaline Phosphatase; ECLIA, Electrochemiluminescence Immunoassay; n.a., not applicable; DL, Detection limit

**Table 6:** HCP content and BAP enzymatic and immunological activities of various drug substance batches.

	**Drug substance**	**Purification step 3**		
	HCP ELISA	Protein	BAP ECLIA	BAP Enzymatic activity
	*[ng/mg]*	*[mg/mL]*	*[U/mg]*	*[mU/mg]*
Batch 3	15	6.61	25	0.3
Batch 4	32	5.78	17	0.3
Batch 5	22	5.70	19	0.3
Batch 6	30	6.13	20	0.2
Batch 7	19	6.23	18	0.2
Batch 8	20	6.56	13	< QL
Batch 9	19	6.40	18	0.2
Batch 10	32	7.57	27	0.4
Batch 11	36	7.42	18	0.3
Batch 12	8	6.78	22	0.2

BAP, Bacterial Alkaline Phosphatase; ECLIA, Electrochemiluminescence Immunoassay; QL, Quantification limit.

## Discussion

For the identification and quantification of HCPs in biotherapeutics, a test system employing HCP affinity enrichment, highly sensitive LC-MS, and high throughput immunoassay testing was developed. Direct LC-MS analysis was capable of identifying 76 HCPs at purification step 1 level but did not verify the presence of HCPs at the final drug substance level. In contrast, the application of affinity chromatography for HCP enrichment combined with LC-MS was adequate to identify 85 HCPs at purification step 1 level. In addition, the results achieved do not suggest, from a qualitative point of view, significant alterations of the HCP profile related to the affinity enrichment step. The described approach resulted in the confirmation of 12 low abundant HCPs in the final drug substance. All identified bacterial HCPs have the potential to trigger an immune response in human, although the respond will depend on composition and amount of bacterial proteins administered [2,3]. Moreover, five low abundant bacterial proteins with possible catalytic activity were verified. 

Bacterial Alkaline Phosphatase (BAP) is a widely distributed non-specific phosphomonoesterase that also catalyzes phosphoryl transfer reaction to various alcohols [31,32]. *E. coli* Alkyl hydroperoxide reductase (AhpC) directly reduces organic hydroperoxides in its reduced dithiol form and might act as an antioxidant enzyme [33]. 2-amino-4-hydroxy-6-hydroxymethyldihydropteridine pyrophosphokinase, Adenylate kinase, and N-acetylmuramoyl-L-alanine amidase AmiD were also detected at the final drug substance level. The identified bacterial enzymes are involved in folic acid biosynthesis, nucleotide biosynthesis, and cell wall biogenesis/degradation [34–37]. The described catalytic activities do not suggest a negative impact on product stability. However, bacterial enzymes could theoretically also have enzymatic effects in humans, although, to the author’s knowledge, reports to this risk have not been published [3]. BAP, AhpC, and Adenylate kinase were detected at purification step 1 and final drug substance level whereas 2-amino-4-hydroxy-6-hydroxymethyldihydropteridine pyrophosphokinase and N-acetylmuramoyl-L-alanine amidase AmiD were only detected at the final drug substance level. Thus, the data indicate a less effective removal or relative enrichment of both enzymes through the purification process.

Subsequently, we focused on developing test systems for high throughput monitoring of specific bacterial HCPs. Since BAP was identified as being the most abundant HCP at purification step 1 level, we firstly established two different assays for enzymatic and immunological BAP monitoring using the cobas® technology. The obtained results demonstrate an almost complete removal of BAP enzymatic and immunological activity by the applied purification process. However, since BAP enzymatic activity was verified until purification step 3 level BAP activity at the final drug substance level is most likely, although to a much lower extent. These results are in agreement with the data obtained by comprehensive LC-MS analysis, in which BAP at final bulk level was only found after enrichment by affinity chromatography. In addition, we assessed if BAP does contribute to the elevated total HCP levels of some drug substance batches. The results revealed that no causal relationship between elevated total HCP levels and enzymatic and immunological BAP activity is traceable. Currently, we are developing novel immunological and enzymatic cobas® test systems to assess AhpC, 2-amino-4-hydroxy-6-hydroxymethyldihydropteridine pyrophosphokinase, Adenylate kinase, and N-acetylmuramoyl-L-alanine amidase AmiD removal by the applied purification process and perhaps more importantly, their catalytic activity at the final drug substance level. Recently, the identification and quantification of HCPs in biotherapeutics by 2D–LC-MS was demonstrated [29,30]. The described approaches allow the simultaneous quantification of various low abundance HCPs. In the present study, the application of affinity chromatography combined with comprehensive LC-MS analysis and cobas® technology was adequate to identify low abundant HCPs at final drug substance level and to subsequently monitor the enzymatic and immunological activity of BAP at various purifications steps. Accordingly, the impact of fermentation, purification, and formulation conditions on HCP removal and biological activity can be conducted by monitoring process-specific HCPs in a GMP-compatible and high-throughput (> 1000 samples/day) manner.

## Materials and Methods

### Reversed Phase Chromatography (RP-HPLC)

Reversed phase chromatography was performed on a Dionex Summit^®^ HPLC system (Thermo Fisher Scientific, Bremen, Germany) with UV detection at 210 nm. The separation was carried out on a YMC-Pack ODS-AQ analytical column (3 x 150 mm, S-3μ, 200 Å, carbon content: 11.0-11.5 %, YMC, Tokyo, Japan) between 20°C and 25°C. A step gradient using 0.1% TFA, 30 % acetonitrile as solvent A and 0.1 % TFA, 80 % acetonitrile as solvent B at 0.4 mL/min was applied. For the chromatographic analysis 6 µg of total protein was injected. Fractions were collected manually.

### Sodium dodecyl sulfate polyacrylamide gel electrophoresis (SDS-PAGE)

One-dimensional SDS-PAGE was performed with Novex® 18% Tris-Glycine gels in a XCell SureLock® Mini-Cell (Life Technologies Corporation, Darmstadt, Germany). Samples were reduced with NuPAGE® Sample Reducing Agent (catalogue number: NP0004) and gel electrophoresis was carried out according to the Novex® Tris-Glycine Midi Gels instruction (quick reference card, 25-0913 Version B; 12 May 2008). 5 µg of protein was loaded per lane. Low concentrated fractions from reverse phase chromatography were evaporated to dryness in a RVC 2-25 CD plus Rotational-Vacuum-Concentrator (Martin Christ Gefriertrocknungsanlagen GmbH, Osterode am Harz, Germany). The gels were stained with SimplyBlue™ SafeStain (Life Technologies Corporation) and destained in ultrapure water. 

### In-gel proteolytic digestion

In-gel digestion was carried out using OMX-S® devices (OMX GmbH, Martinsried, Germany) according to the OMX-S® pro Instruction Manual. Protein bands were washed and destained with 50% acetonitrile, 50% ammonium bicarbonate solution (50 mM, pH 8.0). The digest was performed in 20 µL freshly prepared trypsin solution (0.01 µg/µl) in 50 mM ammonium bicarbonate solution (pH 8.0) at 37°C for 45 min. 

### HCP identification by Nano ESI-MS/MS

For the Nano ESI-MS/MS analysis of the in-gel digests, peptides were desalted and concentrated with ZipTip® C18 Pipette Tips (Millipore Corporation, Billerica, USA) and eluted in 20 µL 1% formic acid, 50% acetonitrile. Nano ESI-MS/MS analyses were performed on a QTOF Ultima mass spectrometer (Waters, Manchester, U.K.) equipped with a TriVersa NanoMate (Advion, Inc., Ithaca, USA). Spectra were recorded in the positive ion mode. Sequencing was performed by low-energy collision-induced dissociation (CID) using argon as collision gas. The collision energy was set from 20 to 45 eV. Spectra were searched against the UniProt database of *Escherichia coli* using Protein Prospector (UCSF, University of California, San Francisco; http://prospector.ucsf.edu/prospector/mshome.htm) and MASCOT (Matrix Science Inc, Boston, USA; http://www.matrixscience.com/search_form_select.html).

### In-solution digestion

For the proteolytic digestion, samples were denatured in 0.4 M Tris-HCl, 8 M guanidine hydrochloride, pH 8.5 by diluting 150 µg of protein in a total volume of 300 µL. For reduction, 10 µl of 0.1 g/mL dithiothreitol was added followed by incubation at 50°C for 1 hour. After alkylation of free cysteine by adding 10 µl of 0.33 g/mL iodoacetic acid and incubation at room temperature under exclusion of light for 30 min, the buffer was exchanged to digestion buffer (0.1 M Tris-HCl, pH 8.5) by application onto an illustra™ NAP™-5 gel filtration column (G-25, GE Healthcare, Buckinghamshire, UK). The NAP™-5 eluate (500 µL) was mixed with 30 µL of a solution of 0.1 mg/mL sequencing grade trypsin (Promega, Madison, USA) in 10 mM HCl and incubated at 37°C for 18 h.

### HCP identification by LC-MS/MS

LC-MS/MS analysis of in solution digests were performed on a LTQ Orbitrap Velos mass spectrometer (Thermo Fisher Scientific, Bremen, Germany) coupled to an Acquity UPLC system (Waters, Manchester, UK). Peptides were separated on a BEH C18 column (1.7 µm 2.1x150 mm, Waters, Manchester, UK) using a binary gradient (solvent A: 0.1% formic acid, solvent B: 0.1% formic acid, 100% acetonitrile) from 1% to 45% B at a flow rate of 0.3 mL/min in 40 min. 5 µg of digested protein was loaded. Low concentrated digests were evaporated to 30 µL in a RVC 2 - 25 CD plus Rotational-Vacuum-Concentrator and completely injected. Data acquisition was controlled by XCalibure software (Thermo, Waltham, MA). For the top10 CID method, survey full scan MS spectra (from m/z 200 - 2000) were acquired by the Orbitrap system with a resolution of r = 30000. The ten most abundant peptide ions with charge states > +1 were sequentially isolated and fragmented with CID and a normalized collision energy of 40 V using helium as collision gas. The resulting fragment ions were detected by the ion trap. The automatic gain control (AGC) target values were set to 1*10^6^ for Full MS scans in the Orbitrap mass analyzer and 1*10^4^ for MS/MS scans in the ion trap mass analyzer.

### LC-MS/MS database query

CID spectra were searched against an in-house *E. coli* database using Proteome Discoverer V1.3 (Thermo Fisher Scientific, Bremen, Germany). The custom protein database was compiled from 5,967 *E.coli* protein sequences (from actual Swiss Prot K12 database), the sequence of the therapeutic protein, and the sequence of porcine trypsin. The following SEQUEST® search parameters were applied: (1) trypsin with a maximum of one missed cleavage, (2) carboxymethylation of cysteines as fixed and (3) oxidation of methionine as variable modifications. Precursor mass tolerance was set to 10 ppm and the fragment mass tolerance was set to 0.8 Da. A False Discovery Rate (FDR) of 1% was calculated. Only proteins specified with a score value ≥ 20 and with high conﬁdence unique peptides (FDR < 1%) were considered as positive hits. The mass spectrometry proteomics data for HCP identification at drug substance level have been deposited to the ProteomeXchange Consortium

(http://proteomecentral.proteomexchange.org) via the PRIDE partner repository[38,39] with the dataset identifier PXD000509 and DOI 10.6019/PXD000509.

### HCP enrichment by affinity chromatography

For the preparation of the affinity resin, NHS activated sepharoseTM 4 fast flow (GE Healthcare, Buckinghamshire, UK) and anti-*E. coli* antibody (Dako Deutschland GmbH, Hamburg, Germany) were used. The antibody solution was dialyzed against coupling buffer (200 mM potassium phosphate, pH 8.4) and adjusted to a concentration of ca. 10 mg/mL. Coupling was performed with an adapted procedure derived from technical note 71 5000 14 AD (GE Healthcare, Buckinghamshire, UK). Unspecific binding sites of the resin were blocked by incubating with drug substance solution. The prepared affinity resin was filled into a Kronlab Eco glass column (10 x 250 mm, YMC Europe GmbH, Dinslaken, Germany) and regenerated by the following steps: (1) 0.5 M NaCl, 0.05 % Tween20, pH 7.5, 4 CV (column volumes); (2) 30 mM NaCl, 4 CV; (3) 1 M propionic acid, 2 CV; (4) 50 mM potassium phosphate, 150 mM NaCl pH 7.5, 2 CV. 2.2 mg of drug substance solution containing 80 µg HCP were loaded on the affinity resin. Column equilibration was performed with 10 mM potassium phosphate, 154 mM NaCl, 0.005 % NaN_3_, 0.05 % Triton-x 100, 0.1 % BSA pH 7.0, 4 CV followed by washing step 1 with 10 mM potassium phosphate, 100 mM NaCl, 0.005 % NaN_3_, 0.05 % Tween20, 0.5 mM EDTA, pH 6.5 (4 CV) and washing step 2 using 10 mM potassium phosphate, 30 mM NaCl, pH 7.5 (4 CV). HCPs were eluted with 3 CV of 1 M propionic acid. Flow rate was set to 0.15 ml/min. The eluate was collected as a single fraction in potassium phosphate (pH 7.5) and was adjusted to pH 7.5. To remove propionic acid the eluate was dialyzed against 10 mM potassium phosphate, 154 mM NaCl, pH 7.5.

### Quantitative determination of the enzymatic activity of alkaline phosphatase

The colorimetric assay was carried out on a cobas INTEGRA® 400 plus system using the ALP IFCC Gen.2 cassettes (Roche Diagnostics Ltd., Basel, Switzerland) according to the manufacturer's guidelines. Briefly, the principle of the assay is the cleavage of p-nitrophenyl phosphate by alkaline phosphatase into phosphate and p-nitrophenol. The p-nitrophenol released is directly proportional to the catalytic alkaline phosphatase activity. The activity is determined by measuring the increase in absorbance at 409 nm. The assay is performed at 37°C. A minimal sample volume of 250 µL was required. Calibration was done with Bacterial Alkaline Phosphatase (Life Technologies GmbH, Darmstadt, Germany, Catalogue number 18011-015).

### Bacterial Alkaline Phosphatase (BAP) ECLIA

The immunological BAP content was determined by an electrochemiluminescence immunoassay (ECLIA) on a cobas e 411 analyzer (Roche Diagnostics Ltd., Basel, Switzerland) according to the manufacturer's guidelines. Briefly, the assay is based on a sandwich principle using a mouse monoclonal anti-BAP antibody (in-house development). First, 30 µl of sample, biotinylated anti-BAP capture antibody and ruthenium-labeled anti-BAP detection antibody were incubated for 9 min forming a ternary complex. In a second step after addition of streptavidin-coated microparticles, the complex is bound to the solid phase via interaction of biotin and streptavidin during 9 min of incubation. The reaction mixture is aspirated into the measuring cell where the microparticles are magnetically captured to the surface of the electrode. Voltage-induced chemiluminescence is measured by a photomultiplier. The concentration of BAP in the test sample is finally calculated from a BAP (Life Technologies GmbH, Darmstadt, Germany, Catalogue number 18011-015) standard curve of known concentration. 

## Supporting Information

Table S1 **Repeatability of HCP identification results.** Database query was conducted by analyzing LC-MS/MS CID spectra using Proteome Discoverer V1.3 and a false discovery rate FDR < 1% of three purification step 1 elution pool samples (three independent sample preparations). The Top 15 HCPs were sorted according to the score value of Analysis 1. Accession number, http://www.uniprot.org/; MW, theoretical molecular weight.
(XLSX)
